# Advanced Functional Nanomaterials for Energy Conversion and Storage

**DOI:** 10.3390/molecules31111858

**Published:** 2026-05-28

**Authors:** Yongxiao Tuo, Xiaohui Sun

**Affiliations:** 1 Shandong Key Laboratory of Advanced Electrochemical Energy Storage Technologies, College of New Energy, China University of Petroleum (East China), Qingdao 266580, China; 2 College of Carbon Neutrality Future Technology, China University of Petroleum (Beijing), Beijing 102249, China

## 1. Introduction

Energy plays a crucial role in the sustainable development of our economy and society. The transition from traditional fossil fuels to clean and renewable energy sources is vital to mitigate environmental pollution and reduce greenhouse gas emissions. In this context, advanced functional nanomaterials have emerged as promising candidates due to their unique mechanical, electrical, and optical properties, offering distinct advantages for energy-related applications.

This Special Issue focuses on the recent progress in the preparation, characterization, and utilization of nanomaterials for sustainable energy conversion and storage. It encompasses both original research and computational insights, reflecting the diverse approaches used to address energy and environmental challenges.

## 2. Significant Advances in This Research Topic

- **Catalytic Materials for Energy Conversion:** Advanced electrocatalysts are essential for clean energy conversion. Wang et al. [Contribution 1] developed an efficient, low-cost, and platinum-group-metal-free catalyst by growing bimetallic Mn-Mo oxide nanoparticles on nitrogen-doped carbon nanotubes. The resulting material exhibited catalytic activity comparable to commercial Pt/C for the oxygen reduction reaction, with superior durability. The study demonstrates the effectiveness of nitrogen doping and synergistic bimetallic interaction in lowering charge transfer resistance and creating active sites for renewable energy systems.
- **Nanomaterials for Electrochemical Energy Storage:**

To meet the growing demand for high-performance supercapacitors, Duan et al. [Contribution 2] synthesized flower-like NiSb/NiSe nanomaterials on nickel foam using a one-step microwave method. The resulting electrode material showed a high specific capacity and excellent cycling stability. Furthermore, an assembled hybrid supercapacitor with activated carbon achieved a high energy density, highlighting its potential for efficient energy storage.

In another study related to energy storage, Yang et al. [Contribution 3] reported the preparation of nitrogen and sulfur co-doped carbon nanosheets within restricted spaces using a vermiculite template method to enhance lithium-ion capacitance performance.

- **Nanomaterials and Simulation for Energy-Related Applications:**

Understanding interfacial phenomena and adsorption processes at the nanoscale is critical for advancing energy systems. Duan et al. [Contribution 4] used grand canonical Monte Carlo and density functional theory to investigate the effect of various functional groups on water adsorption in activated carbon for heat energy storage. Their findings reveal that functional groups enhance water adsorption capacity by forming hydrogen bonds, with -SO_3_H exhibiting the best performance at low pressures, which provides atomistic insights for optimizing adsorption heat storage materials.

Additionally, Li et al. [Contribution 5] employed non-equilibrium molecular dynamics simulations to study membrane distillation in nanopores, analyzing the influence of different salt solutions on wettability, liquid entry pressure, and flux. The study provides valuable insights into mass transport and wetting behavior, which are useful for energy-saving desalination and related purification processes.

## 3. Conclusions

In summary, the contributions compiled in this Special Issue highlight the versatility and significance of advanced functional nanomaterials in modern energy conversion and storage technologies. From the design of low-cost, high-performance electrocatalysts and supercapacitor electrodes to advanced atomistic simulations of interfacial adsorption and mass transport, these studies demonstrate universal identification rules and deep mechanistic insights that extend far beyond single-reaction systems. We anticipate that these findings will inspire future cross-disciplinary research and accelerate the development of sustainable, clean energy solutions.

